# Outbreak of Panton-Valentine Leukocidin–Associated Methicillin-Susceptible *Staphylococcus aureus* Infection in a Rugby Team, France, 2010–2011

**DOI:** 10.3201/eid2201.150597

**Published:** 2016-01

**Authors:** Elodie Couvé-Deacon, Anne Tristan, Nathalie Pestourie, Christian Faure, Valérie Doffoel-Hantz, Fabien Garnier, Frédéric Laurent, Gerard Lina, Marie-Cecile Ploy

**Affiliations:** University of Limoges, Limoges, France (E. Couvé-Deacon, N. Pestourie, F. Garnier, M.-C. Ploy);; Institut National de la Santé et de la Recherche Médicale, Limoges (E. Couve-Deacon, N. Pestourie, F. Garnier, M.-C. Ploy);; Centre Hospitalier Universitaire Laboratoire de Bactériologie-Virologie-Hygiène, Limoges (E. Couvé-Deacon, N. Pestourie, F. Garnier, M.-C. Ploy);; Hospices Civils de Lyon, Lyon, France (A. Tristan, F. Laurent, G. Lina);; National Reference Center for Staphylococci, Lyon (A. Tristan, F. Laurent, G. Lina);; University of Lyon, Lyon (A. Tristan, F. Laurent, G. Lina);; Cabinet Médical du Midi, Limoges (C. Faure);; Clinique François Chénieux, Limoges (V. Doffoel-Hantz)

**Keywords:** Panton-Valentine leukocidin, methicillin susceptible, *Staphylococcus aureus*, beta lactams, outbreak, abscess, skin, bacterial infections, rugby, sports, athletes, bacteria, toxins, France, staphylococci, MSSA

## Abstract

*Staphylococcus aureus* strains that produce Panton-Valentine leukocidin are known to cause community infections. We describe an outbreak of skin abscesses caused by Panton-Valentine leukocidin–producing methicillin-susceptible *S. aureus* (clonal complex 121) in a professional rugby team in France during July 2010–February 2011. Eight team members were carriers; 7 had skin abscesses.

*Staphylococcus aureus* is a leading cause of community and healthcare-associated infections, notably skin and soft-tissue infections (*1*). A strong epidemiologic link exists between community-associated *S. aureus* and Panton-Valentine leukocidin (PVL), a cytotoxin found particularly in deep primary skin infections (*2*). The prevalence of community-associated methicillin-resistant *Staphylococcus aureus* (CA-MRSA) cases seems to be low but increasing in Europe; however, heterogeneity in prevalence rates among countries occurs (*3*). Many sporadic cases and outbreaks of CA-MRSA infection have been reported in sports teams (*4*), especially among players having regular skin-to-skin contact. We describe an outbreak of recurrent PVL-positive community-associated methicillin-susceptible *Staphylococcus aureus* (MSSA) skin abscesses in a professional rugby team in France.

## The Study

On September 22, 2010, a player (patient 4) was hospitalized for a calf abscess that had spontaneously drained to the skin. Bacterial culture grew PVL-positive MSSA. Investigations identified 3 previous case-patients among the team members during the previous month (Figure). A case-patient was defined as a player on the team who developed a skin abscess. The abscesses began occurring after recruitment of a new player from Fiji (patient 1), who had untreated axillary and back abscesses when he arrived on the team in July 2010. All other case-patients had contact with him during scrimmages, suggesting that cross-transmission occurred by close physical contact. In August 2010, abscesses developed on the left wrist of patient 2 and on the arm of patient 3 (Figure).

**Figure:**
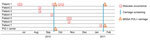
Timeline of infection and screening for PVL-positive *Staphylococcus aureus* case-patients and carriers. MSSA, methicillin-susceptible *S. aureus*; PVL, Panton-Valentin leukocidin; +, positive.

On September 28, 2010, we screened all team members for PVL-positive *S. aureus* carriage. The team had 51 men, including 30 permanent team members; mean age was 23.6 (range 17–42) years. Screening consisted of nasal, throat, and skin-lesion swabbing. *S. aureus* was detected by bacterial culture. Gene-encoding PVL was tested by real-time PCR (*5*). DNA microarray analysis was performed by the French National Reference Center for Staphylococcal Infections (Lyon, France) and enabled detection of the *mecA* gene and genes encoding various toxins and also assisted in *agr* typing and multilocus sequence typing.

The first round of screening showed that 35 (68.6%) of the 51 team members were colonized with MSSA, and 2 (patient 1 and a player who never developed an abscess) harbored PVL-encoding genes (Figure). During the screening process, patient 1 was found to have a PVL-positive MSSA left knee abscess that spontaneously drained to the skin but was not covered.

To reduce risk of transmission, we implemented a 5-day course of *S. aureus* decontamination for all team members; decontamination consisted of mupirocin 2% nasal ointment twice daily and showering with chlorhexidine soap. We also provided information about standard hygiene measures: showering and handwashing; washing jerseys after play; regularly cleaning and disinfecting showers and shared sports equipment; avoiding sharing of personal items; protecting and disinfecting skin lesions; and treating abscesses early and appropriately. Team staff regularly checked players’ adherence to the control measures. Despite these measures, 3 new skin abscesses developed in October 2010 on the chest and nape of patient 1 (Figure; isolates not available). In early November 2010, abscesses developed on the axillary and thigh of patient 5 and on the right wrist (sample not obtained) of patient 6. In January 2011, a thigh abscess developed on patient 7. On February 2, 2011, we conducted a second round of *S. aureus* carriage screening and a 10-day course of *S. aureus* decontamination for 8 players, focusing on previous PVL-positive MSSA carriers and those with abscesses. The screening showed that patients 1 and 7 carried PVL-positive MSSA. All team members were again reminded of basic hygiene measures. These infection control measures were successful: no further person-to-person transmission occurred.

Overall, 8 (15.6%) of the 51 players carried PVL-positive MSSA (n = 3) or had abscesses (n = 7); 2 players had both. All strains isolated in patients 1–7 belonged to clonal complex (CC) 121 and harbored *agr*4 allele. One player who never had an abscess carried a different PVL-positive MSSA strain in his throat (*agr*1, sequence type 152). All isolated PVL-positive MSSA strains were susceptible to all antimicrobial drugs tested except penicillin G. Except for the first 2 abscesses in patient 1, all abscesses were treated with synergistin A and B (2 grams/day for 7 days) and local disinfection.

Using a standardized questionnaire to interview the 51 rugby players, a member of the Hygiene unit at the Limoges Teaching Hospital collected epidemiologic data on demographics, sport practices, sport hygiene, and occurrence of hospitalization or abscess during the previous year. The interviews highlighted poor hygiene practices: 49% of players shared personal items, and fewer than half disinfected or protected skin lesions (Table). Occurrence of abscess during the previous year was the only significant (p = 0.00028, Fisher exact test) risk factor found for a PVL-positive MSSA carriage or abscess; however, given the context of this abscess outbreak, this factor was considered a bias, not a general characteristic.

**Table:** Characteristics of rugby team members involved in outbreak of Panton-Valentine leukocidin–associated methicillin-susceptible *Staphylococcus aureus* infection, France*

Characteristic	Players’ response	Players, no. (%), n = 51	*S. aureus* PVL-positive abscess or carriage,† no., n = 8	Non–*S. aureus* PVL-positive abscess or carriage,† no., n = 43	p value‡
Sharing of personal items	Yes	25 (49.0)	4	21	1.000
	No	26 (51.0)	4	22	
Disinfection of skin lesions	Yes	25 (49.0)	6	19	0.246
	No	24 (47.0)	2	22	
Skin lesion protection in daily life	Yes	18 (35.3)	3	15	0.717
	No	32 (62.7)	5	27	
Skin lesion protection during sport	Yes	24 (47.0)	3	21	0.702
	No	25 (49.0)	5	20	
Hospitalization during previous year	Yes	7 (13.7)	2	5	0.300
	No	44 (86.3)	6	38	
Skin abscess during previous year	Yes	4 (7.8)	4	0	2.8 × 10^−4^
	No	47 (93.2)	4	43	
Body mass index, mean			31.40	27.80	0.057

*PVL, Panton-Valentine leukocidin. †Values are numbers except for body mass index, which is the mean for 51 players. ‡p values used Fisher exact test except for body mass index, which used Student *t*-test. For that category, 95% CI was –0.14 to 7.51.

Skin and soft-tissue infections are common in athletes, and the most common bacterial pathogen responsible for outbreaks is CA-MRSA, particularly the USA300 clone. The strain in the outbreak we investigated was unrelated to USA300 but belonged to CC121. Outbreaks of PVL-positive MSSA skin infections have been described in families in Italy (*6*), schoolchildren in Switzerland (*7*), French soldiers in Côte d'Ivoire (*8*), and prison inmates in France (*9*) . As in this outbreak among rugby players, infection control measures and *S. aureus* decontamination successfully interrupted transmission in most published outbreaks.

In our study, abscess occurred in 4 players despite a round of decontamination strategies. This failure was likely because of an uncovered, untreated knee abscess in the index case-patient (patient 1) during the decontamination period. The continued occurrence of infections highlights the necessity of strict application of hygiene measures.

Nasal carriage of PVL-positive MSSA was not systematically linked to infection (e.g., 1 team member carried a PVL-positive strain in his throat but had no active skin infection). Concordance of skin and soft-tissue infection and nasal carriage is reportedly lower in MSSA than MRSA strains (*10*). Following France’s guidelines for grouped cases of community-associated *S. aureus* infections (http://www.hcsp.fr/explore.cgi/avisrapportsdomaine?clefr=453), we decontaminated all athletes, even those team members not carrying PVL-positive MSSA. Decontamination temporarily reduces risk of colonization of noncarriers. Along with reinforcement of simple personal hygiene measures, our decontamination regimen sufficiently halted transmission without needing to exclude players with abscesses from the team, an important factor in professional sports.

The outbreak strain was *agr*4, PVL positive, and CC121. It belonged to a PVL-positive MSSA lineage that predominated in France during 1981–1990 (*11*). The CC121 *agr*4 lineage was also linked to furunculosis in a study in Poland (*12*). Like the strain in our study, this lineage carried no exfoliative toxin genes and expressed the *seb* superantigen. However, the strain circulating among the rugby team players was positive for *seg*, *sei*, *sem*, *sen*, *seo*, and *seu*. Superficial, deep-skin, and soft-tissue infections linked to CC121, PVL-positive MSSA strains have been reported worldwide (*13*). Similar strains have also been reported in highly lethal community-acquired pneumonia and in severe sepsis with progressive and metastatic soft-tissue infections (*14**,**15*).

## Conclusions

We investigated an outbreak of skin abscesses caused by a PVL-producing MSSA strain and cross-transmitted through physical contact among players of a professional rugby team. A 10-day period of *S. aureus* decontamination combined with reinforcement of hygiene education and practices successfully interrupted person-to-person transmission and enabled control of the outbreak.
